# The Book World of Medicine and Science

**Published:** 1900-08-25

**Authors:** 


### The Book World of Medicine and Science.

Twentieth Century Practice of Modern Medical Science. In 20 volumes. Vol. XIX., "Malaria and Micro-Organisms." (London: Sampson Low, Marston, and Co. 1900.)

This volume is entirely occupied with the consideration of two subjects, the first 500 pages being given up to malaria and the 300 remaining being devoted to a systematic description of the various kinds of micro-organisms which are engaged in the production of disease, including a carefully written essay on the principles and the technique of bacteriology. The article on malaria by Marchiafava and Bignami, of Rome, is the most recent and probably the most complete treatise on the subject that we possess, and it will be read with great interest by that increasing number of students and practitioners who are beginning to recognise the great importance of the whole subject of malarial diseases. After a careful description of the life cycle of the parasite in man, in which the subject is dealt with in general terms, the different forms of parasite associated with the several varieties of the disease are separately described. Then we come to the life cycle of the parasite in the mosquito and a description of the various hæmosporidia which affect birds and animals. The natural history side of the problem having been fully dealt with, we next come to a full description of what is known regarding the etiology of the disease. But the authors have not allowed themselves to be led by the fascination of the subject of the life history of the parasite to neglect the disease itself, and as soon as the biological question is done with we find a very full and complete description of the various manifestation of malaria in which the subject is treated from an ordinary clinical and descriptive standpoint. This portion of the work will prove interesting and useful even to those who do not care to follow out all the new theories which have lately occupied men's minds in regard to the origin and diffusion of the disease. It will be seen, however, that it is impossible to get entirely away from the parasitic theory, and perhaps nothing shows better how thoroughly theory and practice intertwine and interlock at every point, and how important it is, even on the purely practical side, to get hold of an accurate conception of the origin and processes of a disease, than the constant intervention of what some would call theoretical considerations in all that concerns the diagnosis and treatment of malaria. The elucidation of the continuous or irregular fevers of malarial origin other than those of estivoautumnal type, is described as being dependent not on any separate kind of organism, but upon the individual having been attacked by a series of repeated infections. The opinion formerly held by some medical writers that the febrile type might vary was based upon the fact that we often see quotidian change to a tertian, or a quotidian followed by a double quartan or a quartan or *vice versa*. It is now held, however, that the febrile type is within certain limits constant for each species, and when these apparent changes of type take place it may be shown by an examination of the blood that in some cases the one kind of parasite has disappeared, to be replaced by another, or in others that it is a case of double infection by the same kind of parasite. Thus in the quotidians of tertian origin we find two, andin that of quartan origin three generations of parasites which mature successively, with about a day's interval between them. This way of looking at the matter is of course old enough, but it is all made infinitely clearer by the parasitic theory of the origin of the disease. It is far easier to grasp the idea of several generations of creatures maturing each at its proper date, and thus setting up a succession of like phenomena, than of a series of attacks of the same disease racing round in one's internals and never overtaking one another! From a practical point of view, however, it is in the facilities given for the diagnosis of the pernicious and only slightly intermittent or remittent forms of malaria that the recognition of the parasitic origin of the disease is of most importance. The case of a man who in a malarious district suffers from a distinctly intermittent fever following one or other of the ordinary types presents no particular difficulties in the way of diagnosis. But it is quite otherwise with some of the more malignant types of the disease, in which periodicity is for a time, perhaps, entirely absent, and in which, were it not for the discovery of the parasite, it might be impossible to prevent mistakes in diagnosis. The presentment given in this work of the broad clinical facts of the disease and of their relation to recent discoveries regarding its nature and its parasitic origin is a very full and interesting one, and is sure to be useful to that yearly increasing number of medical men who find it their lot to practice in malarious districts. In regard to the second part of this volume, which is devoted to a consideration of micro-organisms in their relation to disease processes, we need not say more than that, while much of it is of a somewhat technical nature, and thus not of such general interest as the earlier chapters, it will be found very useful both as a guide to those who feel able to undertake such investigations, and as a work of reference for those who can only look respectfully, and from afar, at the interesting proceedings therein described. The article on Micro-organisms and Bacteriology is from the pen of Dr. Simon Flexuer, of Philadelphia, and that on the Protozoa from that of Dr. Eugene L. Opie, of the Johns Hopkins University.

Excelsior, being the Quarterly Magazine of the Royal Asylum, Perth, for April, July, October, 1899, and January, 1900.

It would not be quite fair to criticise this kind of literature in the same spirit in which other magazines are dealt with. The editor has not at his back a staff of well-paid contributors, nor even a multitude of scribblers who will accept a small honorarium if they can only see their articles in type; but these asylum journals must all be taken as evidence of care and activity in other branches of management, and of a desire to do something out of the usual routine that may give pleasure to patients and staff. It is certainly creditable to Dr. Urquhart that he has been able to keep a quarterly magazine going year after year, and it is strange that the good example set by Scotland has been so little followed by England. We can hardly say we like the "ponderously jocular" tone of some of the articles, but other papers are interesting. For instance, "Morocco Bound," "From Another Point of View," and we should not forget the pretty little poem called "Come Away." The author, or authoress, of those lines should be encouraged.

